# Carbon black nanoparticle instillation induces sustained inflammation and genotoxicity in mouse lung and liver

**DOI:** 10.1186/1743-8977-9-5

**Published:** 2012-02-02

**Authors:** Julie A Bourdon, Anne T Saber, Nicklas R Jacobsen, Keld A Jensen, Anne M Madsen, Jacob S Lamson, Håkan Wallin, Peter Møller, Steffen Loft, Carole L Yauk, Ulla B Vogel

**Affiliations:** 1 Health Canada, Environmental and Radiation Health Sciences Directorate, Mechanistic Studies Division, Tunney's Pasture, Ottawa, Canada; 2 National Research Centre for the Working Environment, Copenhagen, Denmark; 3 Univesity of Copenhagen, Department of Public Health, Section of Environmental Health, Copenhagen, Denmark

**Keywords:** Oxidative stress, Genotoxicity, DNA strand breaks, Inflammation, Nanoparticles, Carbon Black

## Abstract

**Background:**

Widespread occupational exposure to carbon black nanoparticles (CBNPs) raises concerns over their safety. CBNPs are genotoxic *in vitro *but less is known about their genotoxicity in various organs *in vivo*.

**Methods:**

We investigated inflammatory and acute phase responses, DNA strand breaks (SB) and oxidatively damaged DNA in C57BL/6 mice 1, 3 and 28 days after a single instillation of 0.018, 0.054 or 0.162 mg Printex 90 CBNPs, alongside sham controls. Bronchoalveolar lavage (BAL) fluid was analyzed for cellular composition. SB in BAL cells, whole lung and liver were assessed using the alkaline comet assay. Formamidopyrimidine DNA glycosylase (FPG) sensitive sites were assessed as an indicator of oxidatively damaged DNA. Pulmonary and hepatic acute phase response was evaluated by *Saa3 *mRNA real-time quantitative PCR.

**Results:**

Inflammation was strongest 1 and 3 days post-exposure, and remained elevated for the two highest doses (i.e., 0.054 and 0.162 mg) 28 days post-exposure (P < 0.001). SB were detected in lung at all doses on post-exposure day 1 (P < 0.001) and remained elevated at the two highest doses until day 28 (P < 0.05). BAL cell DNA SB were elevated relative to controls at least at the highest dose on all post-exposure days (P < 0.05). The level of FPG sensitive sites in lung was increased throughout with significant increases occurring on post-exposure days 1 and 3, in comparison to controls (P < 0.001-0.05). SB in liver were detected on post-exposure days 1 (P < 0.001) and 28 (P < 0.001). Polymorphonuclear (PMN) cell counts in BAL correlated strongly with FPG sensitive sites in lung (r = 0.88, P < 0.001), whereas no such correlation was observed with SB (r = 0.52, P = 0.08). CBNP increased the expression of *Saa3 *mRNA in lung tissue on day 1 (all doses), 3 (all doses) and 28 (0.054 and 0.162 mg), but not in liver.

**Conclusions:**

Deposition of CBNPs in lung induces inflammatory and genotoxic effects in mouse lung that persist considerably after the initial exposure. Our results demonstrate that CBNPs may cause genotoxicity both in the primary exposed tissue, lung and BAL cells, and in a secondary tissue, the liver.

## Background

The use of nanoparticles (NPs) in consumer products and applications continues to rise [1]. In parallel, the potential for NP mediated toxicity is a growing public concern. Many of the unique properties exhibited by NPs increase the likelihood of deleterious biological interactions and subsequently, the risk of adverse health outcomes [2-4]. Understanding the repercussions of inhaling NPs is particularly important because NPs penetrate deeper regions of the lung (e.g., alveoli and pulmonary interstitium) [5,6], are translocated from lung to systemic circulation more readily [7,8], and are cleared from the lungs less effectively [9] than their larger counterparts. As such, there is a great probability of cellular interactions, necessitating investigations of NP-mediated toxicity and risk of health consequences.

Carbon black (CB) has been widely investigated since its use as a benchmark control for *in vivo *toxicological evaluation of diesel exhaust particles and as a model of urban air pollution particulate matter almost three decades ago [10,11]. Since then, CB has become the focus of numerous toxicity studies as well as an important reference material (i.e., Printex 90) [12,13]. CBNPs are reactive oxygen species (ROS) generators as shown in cellular [14,15] and acellular systems [16]. Moreover, inhalation or intratracheal instillation exposures to CBNPs result in large pulmonary inflammatory responses in rodents [17-24], which can greatly exacerbate ROS generation via activation of polymorphonuclear (PMN) granulocytes [25]. As such, it is expected that CBNPs can mediate secondary genotoxicity by means of inflammation and oxidative stress. CBNPs are genotoxic *in vitro*, as shown by increases in DNA base oxidation [26], mutation frequency [26,27], strand breaks [28,29] and micronucleus frequency in lung epithelial cells [30] as well as increases in strand breaks in fibroblasts [31]. However, less is known about the genotoxicity of CBNPs *in vivo*. A few studies in rats have demonstrated CBNP-induced DNA base oxidation [32] and increased mutation frequency [20]. However, rats may not be the most suitable model for exposure to particulates due to their predisposition to particle overload. Studies in mice have demonstrated DNA strand breaks in BAL cells [21,33] and one study has established CBNP-induced lung DNA strand breaks, but this was found using a high dose (i.e., 0.2 mg) immediately (3 hours) post instillation [30].

The growing demand for CBNPs for diverse commercial applications (e.g., rubber products and pigments) raises health concerns for the increasing number of individuals routinely exposed, especially in occupational settings where relatively high levels of exposure may occur. As such, it is critical to establish whether genotoxicity and oxidative stress arise *in vivo *at low doses of exposure and in extrapulmonary tissues, and to determine whether these effects are associated with inflammation and/or persist for long periods of time following the initial exposure.

Here, we investigate the relationships between inflammation and genotoxic outcomes over time after a single exposure to Printex 90 CBNPs in BAL cells, lung and liver. Mice were exposed via intratracheal instillation using various doses (i.e., 0.018, 0.054 and 0.162 mg) and post-exposure recovery time-points (i.e., 1, 3 and 28 days), alongside sham controls. We report that instillation of CBNPs leads to prolonged generation of DNA damage in BAL cells, lung and liver of exposed mice as well as persistent pulmonary inflammation, acute phase response and oxidatively damaged DNA.

## Results

### Particle characterization

**Figure 1:**
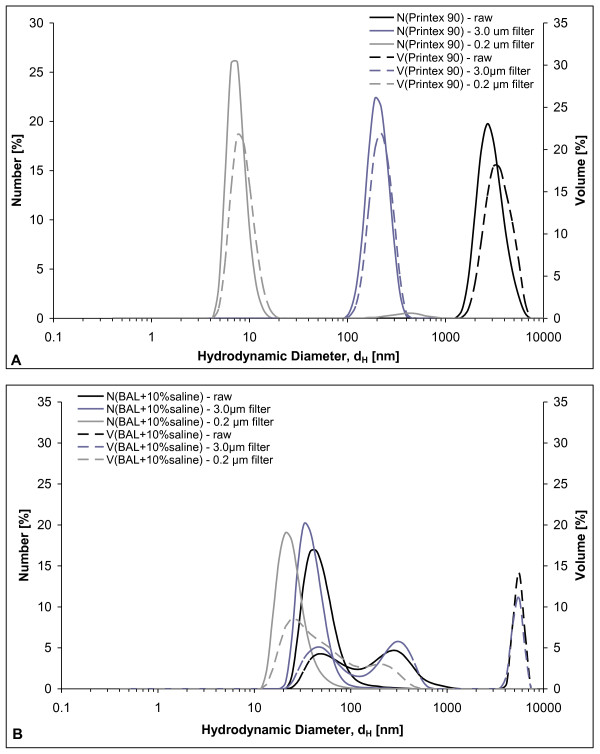
**Dynamic light scattering analysis of instillation medium: A) with 0.054 mg dose of Printex 90 CBNPs and B) instillation medium alone (BAL + 10% saline)**.

Printex 90 CBNPs were a gift from Evonik/Degussa (Frankfurt, Germany). The manufacturer reported an average primary particle size of 14 nm and an organic impurity content of less than 1%. The specific surface area was determined to be 295-338 m^2^/g, corresponding to a theoretical average spherical particle size of 8.1-9.5 nm [34]. The total carbon content measured was greater than 99 wt%, with 0.82 nitrogen and 0.01 hydrogen wt%. Very low levels of both total polycyclic aromatic hydrocarbon (PAH) (74.2 ng/g) [26,27] and total endotoxin (0.142 EU/mg Printex 90) were detected in the sample. The particle suspension (the 0.054 mg dose) was characterised by transmission electron microscopy (TEM) and dynamic light scattering (DLS) analysis as previously reported [34]. The TEM analysis of the instillation suspension showed that the Printex 90 consisted of both free and open to partially open chain-agglomerates (Figure 1A). The size of the primary carbon black spheres was wide, ranging from less than 10 nm to more than 500 nm, whereas the agglomerate sizes typically were on the order of 200 nm or larger. High-resolution analysis showed that the spheres consisted of concentric layers with graphitic spacing, resembling the nano-onions structure.

**Figure 2:**
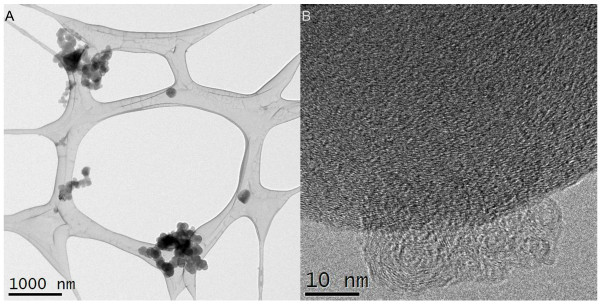
**Transmission electron microscopy images of CBNP particles in the instillation medium**. A) Example of free and agglomerated CBNP spheres of various sizes ranging from nano- to μm-size. Particles are caught on lacy-carbon Cu-grids. B) High-resolution image showing the presence of 10 nm-size CB particles at the rim of a larger CB particle with mottled concentric layers with graphitic structure.

By DLS, the unfiltered Printex 90 instillation suspension (which was employed for intratracheal instillation), was shown to be highly agglomerated in the 10% BAL-saline instillation medium. The propensity towards agglomeration was in accordance with the low peak zeta-potential of -10.7 mV determined for Printex 90 at 0.3 mg/ml instillation medium (conductivity 13.6 mS/cm). The DLS size analysis of Printex 90 stock dispersion has been reported before [34]. Here, we present the data in more detail (Figure 1A). The Printex 90 stock-dispersion was highly agglomerated and had a very high polydispersity index (PDI = 1). The hydrodynamic number size-distribution analysis showed a major peak at approximately 2.6 μm (n = 4), almost comparable to the peak-size (3.1 μm) in the volume-size-distribution. Filtration was performed through a 3.0 and 0.2 μm filter to investigate the possible presence of smaller particles, which may not be detected by DLS due to the high abundance of large agglomerates. Filtration through the 3.0 μm filter (n = 5) revealed the presence of smaller particles with a number peak size of approximately 190 nm (220 nm by volume). Again the polydispersity index was relatively high (PDI = 0.558) indicating a broad size-distribution. This 190-220 nm size corresponds well with the typical smaller free particle and agglomerate sizes of the Printex 90 (Figure 2A). By filtration through a 0.2 μm filter most of the particles were removed resulting in low intensities and hence only a relatively weak, but consistent DLS signal was obtained on this final filtrate. Repeated analyses showed consistent results, but only one out of the first six analyses of the same sample had acceptable data-quality for sizing. The number size-distribution plot showed a highly dominant size-mode with a primary peak-size at 7 nm. By volume, a larger agglomerate mode was also found at 460 nm, which can explain the relatively high polydispersity index (PDI = 0.515) found in the 0.2 μm filtrate. The 7 nm size-mode obtained in the last filtrate could be due to proteins in the dispersion vehicle (BAL fluid). However, analysis of the pure instillation media did not reveal any peak-sizes smaller than 20 nm (Figure 1B). Therefore, the small size material found in the instillation medium is ascribed to a minor fraction of very small Printex 90 particles, which were indeed observed by TEM (Figure 2B). It should be noted that Printex 90 dispersed in MilliQ-filtered water results in much smaller peak sizes of approximatly 50 to 60 nm [35].

### BAL Fluid Cell Composition

We collected BAL fluid from Printex 90 CBNP instilled mice using three doses (i.e., 0.018, 0.054 and 0.162 mg) and three post-exposure time-points (i.e., 1, 3 and 28 days). The inflammatory response was neutrophil dominated and persisted 28 days post-exposure. The largest cellular influxes were observed on post-exposure recovery days 1 and 3 (Table 1).

**Table 1:** BAL fluid cell counts and distribution of cells by type (%) in C57BL/6 mice 1, 3 and 28 days post-exposure to 0

		Control	0.018 mg	0.054 mg	0.162 mg
**24 h**					
	**Neutrophils x10^3 ^(%)**	7.7 ± 1.7 (9.8)	65.0 ± 20.0 (44.3) **	140.0 ± 28.0 (61.5) **	160.0 ± 18.0 (73.9) **
	**Macrophages x10^3 ^(%)**	53.0 ± 2.5 (73.1)	53.0 ± 6.8 (47.2)	50.0 ± 6.3 (31.4)	37.0 ± 5.8 (16.9) **
	**Eosinophils x10^3 ^(%)**	0.3 ± 0.4 (0.3)	1.1 ± 0.4 (0.8)	3.1 ± 2.1 (1.3)	4.2 ± 2.6 (1.6)
	**Lymphocytes x10^3 ^(%)**	1.5 ± 0.2 (2.2)	1.4 ± 0.6 (0.9)	2.5 ± 0.7 (1.3)	1.7 ± 0.9 (0.8)
	**Total BAL Cells x10^3 ^**	74.0 ± 3.6	130.0 ± 16.7 **	200.0 ± 28.0 **	220.0 ± 24.0 **
**3 days**					
	**Neutrophils x10^3 ^(%)**	3.0 ± 2.3 (2.4)	21.0 ± 5.9 (16.2) **	63.0 ± 4.8 (40.5) **	120.0 ± 13.0 (46.7) **
	**Macrophages x10^3 ^(%)**	56.0 ± 4.2 (83.1)	86.0 ± 12.0 (65.8) *	63.0 ± 6.9 (40.3)	89.0 ± 12.0 (33.4) *
	**Eosinophils x10^3 ^(%)**	0.4 ± 0.6 (0.6)	13.0 ± 5.8 (8.8) **	16.0 ± 4.3 (10.4) **	29.0 ± 9.9 (10.8) **
	**Lymphocytes x10^3 ^(%)**	0.9 ± 0.2 (1.4)	3.8 ± 1.0 (2.9) **	4.3 ± 1.0 (2.8) **	8.4 ± 1.8 (3.3) **
	**Total BAL Cells x10^3 ^**	69.0 ± 6.4	130.0 ± 15.0 **	160.0 ± 22.0 **	270.0 ± 24.0 **
**28 days**					
	**Neutrophils x10^3 ^(%)**	1.2 ± 0.2 (1.2)	3.1 ± 0.5 (3.4) *	15.0 ± 3.1 (10.9) **	50.0 ± 13.0 (29.3) **
	**Macrophages x10^3 ^(%)**	82.0 ± 5.7 (85.6)	73.0 ± 9.4 (77.5)	99.0 ± 15.0 (73.3)	75.0 ± 4.3 (48.4)
	**Eosinophils x10^3 ^(%)**	0.3 ± 0.0 (0.3)	3.4 ± 3.4 (2.8)	0.1 ± 0.1 (0.1)	0.1 ± 0.1 (0.1)
	**Lymphocytes x10^3 ^(%)**	2.1 ± 0.4 (2.2)	3.5 ± 1.2 (4.1)	12.0 ± 2.7 (8.9) **	22.0 ± 6.1 (13.4) **
	**Total BAL Cells x10^3^**	96.0 ± 5.8	93.0 ± 10.0	140.0 ± 21.0 **	160.0 ± 16.0 **

Increased total cell counts in BAL fluid were found for all doses and time-points, except for the lowest dose delivered at the 28 day recovery time-point. The net increase in the total number of cells relative to controls in the BAL fluid by the largest CBNP dose was 1.5 × 10^5 ^cells (95% CI: 0.9 × 10^5 ^- 2.1 × 10^5^), 2.0 × 10^5 ^cells (95% CI: 1.3 × 10^5^- 2.8 × 10^5^) and 0.6 × 10^5 ^cells (95% CI: 0.3 × 10^5 ^- 1.1 × 10^5^) in the mice sacrificed on days 1, 3 and 28, respectively.

The increase in total BAL fluid cell counts was primarily the result of large neutrophil influxes, which were observed at all doses and time-points (Table 1). The net influx of neutrophils relative to controls in the BAL fluid following exposure to the highest dose of Printex 90 was 1.5 × 10^5 ^cells (95% CI: 0.7 × 10^5^-3.2 × 10^5^), 1.2 × 10^5 ^cells (95% CI: 0.8 × 10^5 ^- 1.9 × 10^5^) and 0.5 × 10^5 ^cells (95% CI: 0.3 × 10^5 ^- 0.6 × 10^5^) on post-exposure days 1, 3 and 28, respectively.

The number of macrophages decreased following exposure to the highest dose of Printex 90 on post-exposure day 1, but increased following low and high dose Printex 90 exposures on post-exposure day 3 (Table 1). The number of lymphocytes increased 3 days post-exposure for all three doses of Printex 90. Increased numbers of lymphocytes were also observed on post-exposure day 28 for both the medium and high doses of Printex 90, thus suggesting onset of allergic airway inflammation. On post-exposure day 3, increased eosinophils were observed for all doses, and an increased number of epithelial cells was found at the highest dose of Printex 90.

### Acute phase response

Printex 90 increased the mRNA expression of *Saa3 *in lung tissue on day 1 (all doses), 3 (all doses) and 28 (0.018 and 0.054 doses), whereas there were no effects on the *Saa3 *mRNA expression in the liver (Table 2).

**Table 2:** Expression of *Saa3 *presented as fold-changes over matched control in C57BL/6 mice 1, 3 and 28 days post-exposure to 0

		0.018 mg	0.054 mg	0.162 mg
**Lung**				
	**24 h**	63.7 ± 33.2 **	240.3 ± 75.4 **	298.2 ± 78.0 **
	**3 days**	8.2 ± 4.2 **	23.5 ± 6.7 **	50.5 ± 14.6 **
	**28 days**	1.1 ± 0.4	4.9 ± 2.2 **	21.8 ± 10.1 **
**Liver**				
	**24 h**	1.2 ± 0.5	1.6 ± 0.5	1.3 ± 0.3
	**3 days**	0.7 ± 0.2	0.7 ± 0.2	0.7 ± 0.2
	**28 days**	1.0 ± 0.2	1.5 ± 0.2	1.3 ± 0.2

n = 6 except for the control groups

(n = 22)

### DNA Damage

**Figure 3:**
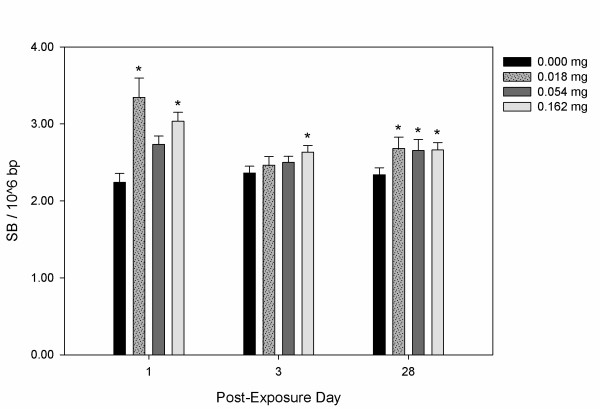
**DNA strand breaks (SB) per 10^6 ^base pairs in C57BL/6 mouse BAL cells following exposure to 0.000, 0.018, 0.054 or 0.162 mg Printex 90 CBNPs and sacrificed 1, 3, and 28 days post-exposure**. Error bars represent the standard error of the mean for 6 mice in each group. *, ** refers to a statistical significance of P < 0.05 and P < 0.001 respectively, when compared by two-factor ANOVA using the day of sacrifice and dose as categorical variables.

We observed increased levels of SB in CBNP Printex 90 instilled mouse lung, liver and isolated BAL cells, relative to sham controls. SB in BAL cells were observed at the high dose (i.e., 0.162 mg) for each time-point, as well as at all doses 28 days post-exposure, relative to controls (Figure 3). DNA SB, even 28 days after exposure, were increased from 2.34 SB per 10^6 ^bp in the control group to between 2.65-2.68 per 10^6 ^bp in the exposed group (i.e., all doses). There was no apparent dose-response relationship.

**Figure 4:**
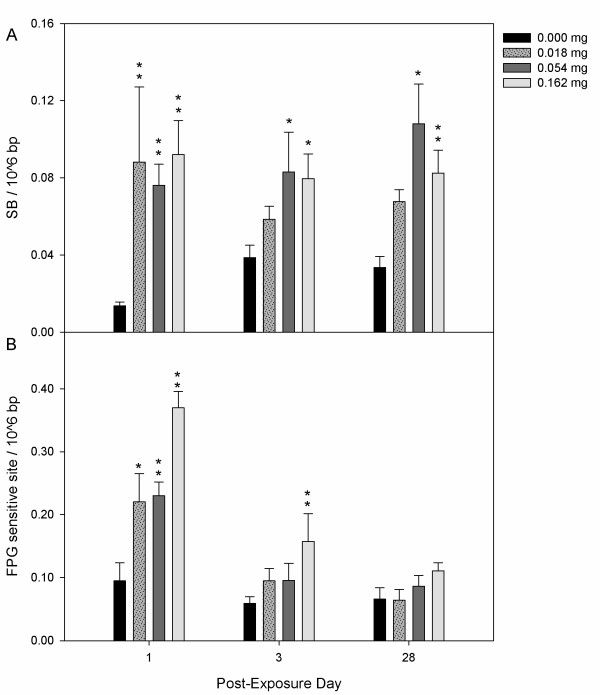
**DNA strand breaks (SB) and formamidopyrimidine DNA glycosylase (FPG) sensitive sites in lung of CBNP exposed mice**. SB (A) and FPG sensitive sites (B) per 10^6 ^base pairs in C57BL/6 mouse lung tissue following exposure to 0.000, 0.018, 0.054 or 0.162 mg Printex 90 CBNPs and sacrificed 1, 3, and 28 days post-exposure. Error bars represent the standard error of the mean for 6 mice in each group. *, ** refers to a statistical significance of P < 0.05 and P < 0.001 respectively, when compared by two-factor ANOVA using the day of sacrifice and dose as categorical variables.

Increased SB were also observed in lung for all doses relative to control, with the largest effects observed on day 1. Levels of DNA SB 24 hours after the exposure were greatly increased from 0.01 SB per 10^6 ^bp in the control group, to between 0.08-0.09 SB per 10^6 ^bp in the exposed group (i.e., all doses) (Figure 4A). Mice exposed to the higher doses (i.e., 0.054 and 0.162 mg) also exhibited elevated levels of SB 3 and 28 days post-exposure relative to control animals.

The number of formamidopyrimidine DNA glycosylase (FPG) sensitive sites in lungs (an indicator of oxidative damage to DNA) was significantly elevated in all dose groups of the CBNP exposed animals relative to controls on day 1, with FPG sensitive sites of 0.22-0.37 per 10^6 ^bp in the exposed animals, compared to 0.10 per 10^6 ^bp in the controls (Figure 4B). Statistically significant increases in FPG sensitive sites were also observed for the high dose exposure (i.e., 0.162 mg) at the 3 day recovery time-point. Marginal increases were observed for the low dose groups on day 3 (i.e., 0.018 and 0.054 mg) and for the high dose groups on day 28 (i.e., 0.054 and 0.162 mg) in a dose-response trend.

**Figure 5:**
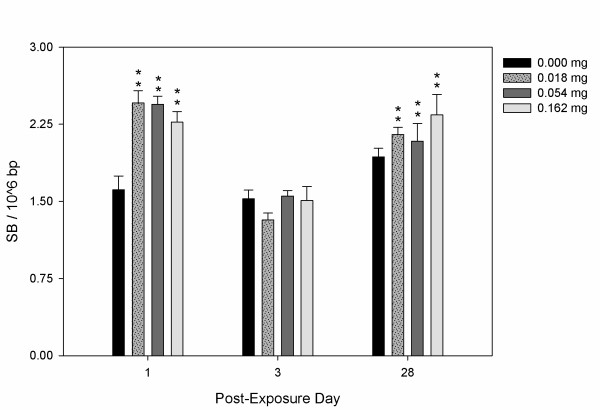
**DNA strand breaks (SB) per 10^6 ^base pairs in C57BL/6 mouse liver following exposure to 0.000, 0.018, 0.054 or 0.162 mg Printex 90 CBNPs and sacrificed 1, 3, and 28 days post-exposure**. Error bars represent the standard error of the mean for 6 mice in each group. *, ** refers to a statistical significance of P < 0.05 and P < 0.001 respectively, when compared by two-factor ANOVA using the day of sacrifice and dose as categorical variables.

DNA damage also occurred in liver of the CBNP exposed mice with significantly elevated levels of SB on days 1 and 28 (Figure 5), although not on day 3. Levels of SB were increased from 1.61 lesions per 10^6 ^bp in the control group, to 2.27-2.46 SB per 10^6 ^bp 24 hours post-exposure and from 1.93 lesions per 10^6 ^bp in the control group, to 2.10-2.34 SB per 10^6 ^bp 28 days post-exposure. No apparent dose-response relationships were observed in the liver.

In the lung, there was a strong correlation (r = 0.88, P < 0.001) between FPG sensitive sites and BAL cell numbers across the doses and time points, whereas no such correlations were observed between the number of BAL cells and SB (r = 0.52, P = 0.08). The correlation between the number of BAL cells and FPG sensitive sites in the lung was mainly driven by the differences related to the doses and time points because the statistically significant association between BAL cell influxes and FPG sensitive sites (r = 0.61, P < 0.001) vanished in regression models adjusted for the effect of dose and time-point.

## Discussion

We investigated CBNP-mediated DNA damage and inflammatory responses in Printex 90 instilled mice using multiple doses (i.e., 0.018, 0.054 and 0.162 mg) and post-exposure time-points (i.e., 1, 3 and 28 days), alongside sham controls. These doses equal the pulmonary deposition after 1, 3 and 9 working days for a mouse at the occupational exposure limit of 3.5 mg/m^3 ^CB per 8 hour work shift (as established by the Occupational Safety and Health Administration (OSHA) and the National Institute for Occupational Safety and Health (NIOSH)). These calculations assume that 33.8% of the inhaled mass ends up in the pulmonary region [33] with a volume of inhaled air per hour of 1.8 L/h [36] and 8 h working days. CB exposure levels of up to 3.7 mg/m^3^, 2.2 mg/m^3 ^and 4.2 mg/m^3 ^have been reported for workers implicated in packaging and in handling CB [37-39]. BAL cell influxes revealed CBNP-induced inflammation that peaked 1 and 3 days post-exposure and persisted 28 days thereafter (Table 1). SB were detected in lung and liver tissues and isolated BAL cells relative to controls and persisted to day 28 (Figures 3, 4A and 5). FPG sensitive sites in lung were increased throughout with significant increases occurring on post-exposure days 1 and 3 in comparison to controls (Figure 4B). The expression of *Saa3 *mRNA in lung tissue was increased at all time-points. This reflects a persistent acute phase response during the entire experiment (Table 2). The correlation between total BAL cells and FPG sensitive sites across all doses and time-points suggests an important role for BAL cell influxes in generation of oxidative stress in the lungs. This study is the first to demonstrate *in vivo *low dose CBNP-induced SB in lung and liver, which persist 28 days after a single exposure.

Although the primary particle size of Printex 90 (as dry powder) was stated by the supplier to be 14 nm, CBNP suspensions were shown to be highly agglomerated by DLS. When analyzing the unfiltered suspension we were only able to detect particles with a peak size of 2.6 μm. However, this is an artefact of the DLS methodology in which larger agglomerated particles will overshadow smaller particles. Consequently, DLS on unfiltered samples can only detect the largest particles in the suspension. Therefore, we filtered the suspension to remove the largest particles in order to be able to detect the smaller particles. DLS on the filtered suspension confirmed the presence of smaller particles as well (peak size of 190 nm and range of 90-350 nm). This shows that some of the particles in the suspension used for instillation are nano-sized, but the DLS analysis is unable to quantify the proportion of nano-sized particles in the suspension.

As in other studies of NP toxicity, endotoxin present on the NPs can result in inflammatory responses. However, the very low level of endotoxin detected on the CBNPs (0.142 EU/mg CBNP) is not anticipated to result in any significant inflammatory response. In another study, the response of LPS instillation was investigated in mice [40]. Instillation of a high dose of LPS (100 μg = 1200 000 EU) resulted in a high inflammatory response, while a low dose of LPS (0.1 μg = 1200 EU) resulted in a low inflammatory response. We have instilled doses ranging from 0.003 EU (0.142 EU/mg × 0.018 mg) to 0.02 EU (0.142 EU/mg × 0.162 mg). The ensuing doses of endotoxin administered in our study are therefore several orders of magnitude below what is considered a low dose of LPS, and thus are expected to result in very little (if any) endotoxin induced inflammation.

SB in lung and BAL cells demonstrate CBNP-induced DNA damage at the site of exposure (Figures 3 and 4A). Although CBNP-induced DNA SB have been investigated *in vitro *[30,31,33,34], only one study has investigated SB in mouse lung *in vivo*, and this work used a single high dose (i.e., 0.2 mg instillation) [30]. In contrast, we demonstrate that SB in lung occur at much lower doses of exposure (e.g., as low as 0.018 mg). Our BAL findings corroborate two earlier studies demonstrating CBNP-induced SB in C57BL/6 and tumour necrosis factor (TNF) deficient mice exposed by inhalation (i.e., 20 mg/m^3^, 90 min/day, 4 days) [21], and apolipoprotein E (ApoE) deficient and C57BL/6 mice exposed by instillation (i.e., 0.054 mg) [33]. Thus, our results indicate that SB result from exposure levels that are much lower than previously reported.

The aforementioned *in vivo *works investigated pulmonary and BAL SB shortly after CBNP exposure, using recovery time-points ranging from 1 to 24 hours [21,30,33]. Taking into consideration that SB are readily repaired within a few hours of induction [41], we aimed to establish whether *de novo *DNA SB could be induced at much later post-exposure time-points (e.g., 3 and 28 days). Interestingly, we observed SB at all post-exposure time-points for the mice exposed to the high dose relative to controls in both lung and BAL cells, and these effects often extended to lower dose groups (Figures 3 and 4A). As such, we demonstrate that CBNPs can generate pulmonary and BAL SB even 28 days after a single exposure. In addition, we demonstrate that assessing SB in cells may serve as a potential biomarker of pulmonary genotoxicity in human studies.

Several mechanisms are hypothesized to contribute to CBNP-induced genotoxicity. It has been suggested that CB may be genotoxic because of adsorbed surface compounds, primarily polycyclic aromatic hydrocarbons (PAHs). However, because of the low levels of PAHs found on the CBNP surface (e.g., 72.4 ng/g in the Printex 90 used in this work) and the high affinity of these compounds for the CB particle core, CBNP-induced toxicity is not likely mediated via PAH exposure [26,27,42]. Alternatively, CBNPs have been identified as potent ROS generators [14-16] and are also associated with large inflammatory responses, as established by our BAL cell profiles and by the work of others [17-24]. Pulmonary influxes in BAL cells induce ROS production at sites of acute inflammation as a result of degranulation and oxidative burst mechanisms [43]. More importantly, the mutation spectrum arising *in vitro *following CBNP exposure points to ROS as the primary mutagenic factor [27], providing substantial evidence that ROS-induced DNA damage occurs upon CBNP exposure. As such, we speculate that oxidative stress could be an important parameter of toxicity requiring further investigation *in vivo*.

It has been established that oxidative damage to DNA leads to increased mutation frequencies, and there is increasing evidence for a direct association between levels of oxidatively damaged guanine lesions and increased risks of lung cancer in humans [44,45]. In contrast to BAL cells that are generated from hematopoietic stem cells, mutations in lung can result in permanent genetic changes within tissue and therefore increased cancer risk. As such, we examined lung tissue directly for oxidative damage to DNA. FPG sensitive sites were quantified using the alkaline comet assay, by which oxidized purines, primarily 8-oxo-7, 8-dihydro-2'-deoxyguanosine (8-oxodG) and 2, 6-diamino-4-hydroxy-5-formamidopyrimidine are recognized. 8-oxodG is the most commonly oxidized lesion and is pro-mutagenic due to its ability to base-pair with adenine, resulting in G to T transversions [46]. Previous *in vitro *work has revealed non-significant increases in the levels of FPG sensitive sites in human Caco-2 cells exposed to CB (20 μg/cm^2^, 4 h exposure) [47], whereas significant increases in FPG sensitive sites were found in an immortalized Muta™Mouse lung epithelial cell line (11.3 μg/cm^2 ^or 75 μg/ml, 3 h exposure) [26]. Here, we demonstrate that CBNPs induce FPG sensitive sites *in vivo *in the lungs. This is consistent with a previous inhalation study in rats where an elevated dose of Printex 90 (i.e. more than 5 mg retained in the lungs) caused increased 8-oxodG, which persisted for 44 weeks [32]. No effects were observed for lower doses. Considering differences between species (e.g., approximate weights of 20 and 200 g and pulmonary surface area of 82.2 and 300 cm^2 ^in mouse and rat respectively [48,49]) this 5 mg retained dose in our model would translate to instilled doses of 0.5 mg or 1.4 mg according to body weight and lung surface area, which is well above our highest exposure dose. However, levels of 8-oxodG can often be elevated by spurious oxidation [50] and thus it is possible that effects may have been detected at lower dose levels with lower background levels. A high dose (i.e., 0.050 mg × 6) of Printex 90 repeated six times in six weeks also caused increased immunostaining for 8-oxodG in the lungs of mice [51]. Thus, in the present study we observed increased FPG sensitive sites at much lower CBNP doses (i.e., 0.018 and 0.054 mg) and at later post-exposure recovery time-points relative to previous studies, with significant increases occurring on post-exposure days 1 and 3 (Figure 4B).

The increase in possible oxidatively damaged DNA observed in our study in comparison to controls was much higher than previous *in vitro *observations. For example, [26] showed a less than two fold increase over controls in lung epithelial cells, following 3 h of exposure to 75 μg/ml CBNPs (a dose of approximately 11.3 μg/cm^2 ^cells compared to 0.2-2.0 μg/cm^2 ^in the current work [48]). This might be because of the presence of ROS-producing granulocytes in the mouse lung, as indicated by the close correlation of PMN cells and FPG sensitive sites found. In keeping with this observation, rats exposed by intratracheal administration of 0.64 mg/kg body weight (i.e., our low dose is approximately 0.9 mg/kg body weight in mouse), developed neither inflammation in terms of PMN infiltration nor increased levels of 8-oxodG in lung parenchyma [52]. We speculate that the pulmonary genotoxocity observed in our work is most likely related to oxidative stress mediated by inflammatory cells, which is in accordance with previous work that has shown CBNP-induced mutation frequency increases only after inflammation is established [20]. On the other hand, we have previously found that SB and pro-inflammatory effects occurred independently of each other *in vitro *[53] and in BAL cells *in vivo *[21,34,54,55]. In the present study, inflammation and BAL cell SB were observed at all doses and time points, and thus, we are not able to determine if the observed SB are caused by inflammation or whether inflammation and genotoxicity occur independently of each other.

Large inflammatory responses, such as the ones observed in our work, are associated with systemic effects of exposure due to increases in circulating inflammatory cells (e.g., PMN) and molecular mediators of inflammation (e.g., pro-inflammatory cytokines and chemokines). Additionally, NPs themselves have been demonstrated to translocate into systemic circulation [56] and spark generated ultrafine carbon NPs have been shown to accumulate in liver of rats upon inhalation [8]. Likewise, individual exposure to traffic related air pollution at the home address and by occupation has been associated with an increased risk of hepatic cancer [57,58]. In order to investigate the possibility of adverse effects in extra-pulmonary tissues, we used the alkaline comet assay to quantify SB in liver tissue of the same mice. We found that SB were elevated on post-exposure days 1 and 28 (Figure 5). The reason for the lack of damage on day 3 remains unclear. It is possible that two separate mechanisms may come into play at different times (e.g., direct instillation effects on day 1 vs. particle relocation to liver or persistent inflammation on day 28), thus not affecting this post-exposure time-point. The results on particle-induced hepatic DNA damage are consistent with our recently published study on DNA damage in mice exposed to Printex 90 by inhalation [35]. Two different mechanisms are hypothesized to be responsible for the observed hepatic DNA damage: 1) direct particle-mediated effect caused by particles translocated from the lungs to the systemic circulation, and 2) indirect effects related to systemic inflammation. To investigate if the hepatic DNA damage resulted from a systemic acute phase response, we measured the mRNA expression of *Saa3 *in pulmonary and hepatic tissue. As we reported before with mice exposed to CB or diesel exhaust particles by inhalation [59], we did not detect a change in *Saa3 *mRNA in the liver in the current study. In contrast, we found a large increase in pulmonary *Saa3 *mRNA expression. SAA protein can be detected in circulation during pulmonary inflammation and may induce a systemic acute phase response [60]. We have recently reported that the acute phase response is also induced in the lungs of mice exposed to nanotitanium dioxide where increased levels of SAA protein was also detected in lung tissue [61]. We do not know whether the hepatic effects are caused by inflammation or direct effects of translocated particles, but since translocation of the CBNPs is expected to be low, and particles accumulate primarily in Kupffer cells in the liver [62], the observed hepatic effects are most likely caused by inflammation. To our knowledge, our work is the first to demonstrate that hepatic SB occur as a result of exposure to CBNPs via intratracheal instillation.

There is increasing evidence that both DNA SB and oxidative damage to DNA can lead to increased risk of tumorigenesis and carcinogenesis [63-66]. Likewise, the relationship linking chronic inflammation to increase risk of carcinogenic outcome is increasingly well supported [67]. As such, it is likely that regular exposure to CBNPs is involved in adverse health outcomes that include cancer. Although previous epidemiological data have linked CBNP exposures to pulmonary carcinogenesis [68-70], evidence to date is insufficient to classify these particles as human carcinogens (currently classified as possibly carcinogenic to humans under Group 2B) [71]. As such, further investigations should elucidate additional underlying mechanisms of toxicity induced by CBNPs and their relationship with disease development. This should include simultaneous investigations of oxidative stress, genotoxicicty and inflammation in lung and liver of exposed mice using multiple endpoints (e.g., micronucleus assay, mutation analysis and GSH levels) and in repeated chronic daily inhalation studies to more closely mimic human exposures. Furthermore, as effects were observed at lower doses of exposure, dose-response relationships should be examined to establish the lowest observable effects and no observable effects levels. In addition to mechanistic studies, future works should clearly establish current human exposure levels in order to evaluate the risks of adverse health effects in individuals routinely exposed to CBNPs.

## Conclusion

CBNP exposed mice exhibited increased DNA SB and inflammation at exposure doses as low as 0.018 mg and as much as 28 days following a single exposure in BAL cells, lung and liver tissue. Oxidative stress conditions were suggested by the presence of FPG-sensitive sites in DNA. The data suggest that these related mechanisms (e.g., inflammation and DNA damage) can interact and possibly contribute to carcinogenic outcomes, potentially at lower doses than suggested previously.

## Materials and methods

### Animals

Female C57BL/6 mice aged 5-6 weeks were obtained from Taconic (Ry, Denmark). Mice were acclimatized for 2-3 weeks before the experiment and were 8 weeks of age at the start of the study. All mice were given food (Altromin no. 1324, Christian Petersen, Denmark) and water *ad libitum*. The mice were group housed in polypropylene cages with sawdust bedding at controlled temperature (21 ± 1°C) and humidity (50 ± 10%) with a 12-h light:12-h dark cycle. The experiments were approved by the Danish "Animal Experiments Inspectorate" (permit 2010/561-1179) and carried out following their guidelines for ethical conduct and care when using animals in research. All procedures complied with EC Directive 86/609/EEC and Danish laws regulating experiments on animals.

### Preparation of exposure stock

Printex 90 was suspended by sonication in 0.9% NaCl MilliQ water containing 10% v/v acellular BAL from C57BL/6 mice. The BAL fluid was prepared by flushing unexposed mice twice with 0.6 ml 0.9% NaCl solution yielding approximately 1 ml of BAL fluid. Acellular BAL was prepared by centrifugation of BAL fluid at 400 g (10 min, 4°C). The particle suspensions (4.05 mg/ml) were sonicated using a 400 W Branson Sonifier S-450D (Branson Ultrasonics Corp., Danbury, CT, USA) equipped with a disruptor horn (Model number: 101-147-037). Total sonication time was 8 min, with alternating 10 s pulses and 10 s pauses at amplitude of 10%. Samples were continuously cooled on ice during the sonication procedure. This suspension was used for the high dose, diluted 1:3 for the medium dose (0.054 mg) and diluted further 1:3 for the low dose (0.018 mg). Vehicle control solutions were prepared containing 90% 0.9% NaCl MilliQ water and 10% acellular BAL fluid.

### Characterization of exposure

The hydrodynamic particle size distributions in the exposure media were determined by Dynamic Light Scattering (DLS) using a Malvern Zetasizer Nano ZS (Malvern Instruments Ltd, UK). Data were analyzed using the Dispersion Technology Software (DTS) version 5.0 (Malvern Instruments Ltd). Samples were measured at 25°C in 1 mL Malvern disposable polystyrene cuvettes. For analysis of hydrodynamic size, we used the refractive (R_i_) and absorption indices (R_abs_) of 2.020 and 2.000, respectively, for Printex 90 and standard optical and viscosity properties for H_2_O. For analysis of the instillation medium we used the Malvern standard conditions for both water and protein (R_i _= 1.450; R_abs _= 0.001). The nature of particles and level of agglomeration was also evaluated by TEM using a 200 kV Tecnai T20 G2 Transmission Electron Microscope at the DTU Center for Electron Nanoscopy, Technical University of Denmark, Lyngby. DLS analysis of exposure media was performed on the raw instillation dispersions and after filtration through 3 μm (Glass Micro Fiber;Whatmann, UK) and 0.2 μm (hydrophilic DISMIC^®^-25CS Cellulose Acetate; Toyo Roshi Kaisha Ltd, Japan) syringe filters. Zeta-potential measurements were completed using instillation mediums with a Printex 90 concentration of 0.3 mg/ml according to the Smoulowkowski model and using Malvern's folded capillary cuvettes DTS1060 in the general purpose mode using the Malvern Zetasizer above. All data were obtained based on six consecutively repeated analyses of the same sample with no pause. Acceptance of the size-distribution data was done after evaluation of the correlation spectra and size-distribution fit and standard quality reports in the software. The quality of the zeta-potential measurements was controlled by evaluation of counts, sample conductivity, phase-plots, as well as zeta- and mobility data. In all cases, outliers identified following quality control were removed from the final average value.

### Exposure of mice

A total of 72 mice (6 per group) were given 0.018, 0.054 or 0.162 mg of Printex 90 CBNPs by a single intratracheal instillation. Before the intratracheal instillation, the mice were anesthetized using Hypnorm^® ^(fentanyl citrate 0.315 mg/ml and fluanisone 10 mg/ml from Janssen Pharma) and Dormicum^® ^(Midazolam 5 mg/mL from Roche). Both anaesthetics were mixed with equal volumes sterile water. A volume of 0.15 ml was injected subcutaneously in the neck of each mouse. The sedated mice were kept on 37°C heating plates. During instillation the mice were placed on their backs on a 40 degree slope. The trachea was intubated using a 24 gauge BD Insyte catheter (Becton Dickinson, Denmark) with a shortened needle. The correct location of each intubation was tested using a highly sensitive pressure transducer (pneutachymeter) developed at the National Research Centre for the Working Environment in collaboration with John Frederiksen (FFE/P, Copenhagen, Denmark). A 40 μl suspension was instilled followed by 150 μl air with a 250 μl SGE glass syringe (250F-LT-GT, MicroLab, Aarhus, Denmark). Control animals received vehicle instillations. The intubation catheter was held head up until proper breathing was assured. The mice were then transferred to the 37°C heating plate until they recovered from anaesthesia. The animals did not show signs of respiratory distress, lethargy or other physical symptoms of exposure.

### Preparation of tissue and cells from the mice

One, 3 and 28 days after the instillation, the mice were anaesthetised with Hypnorm/Dormicum as described above. Immediately after withdrawing the heart blood, a bronchoalveolar lavage (BAL) was performed four times with 0.8 mL of 0.9% sterile saline through the trachea. The BAL was immediately put on ice until BAL fluid and BAL cells were separated by centrifugation at 4°C and 400 g for 10 min. The BAL cells were resuspended in 100 μL medium (HAMF12 with 10% fetal bovine serum). The suspension (40 μL) was mixed with 160 μL medium containing 10% DMSO and stored at -80°C for later analysis in the comet assay. For differential count, cells from 50 μL were collected on microscope slides by centrifugation at 10, 000 rpm for 4 min in a Cytofuge 2 (StatSpin, Bie and Berntsen, Rødovre, Denmark). The slides were fixed with 96% ethanol and stained with May-Grünwald-Giemsa stain. The cellular composition of BAL cells was determined on 200 cells. The total number of cells was determined by using the NucleoCounter (Chemometec, Allerød, Denmark) live/dead assay according to the manufacturer's instructions. The lungs and liver were snap frozen in cryotubes (NUNC) in liquid N_2 _and stored at -80°C.

### DNA Damage measured by the Comet Assay

Whole lung and liver were broken into specific pieces under liquid nitrogen, homogenized in Merchant's EDTA (0.14 M NaCl, 1.47 mM KH_2_PO_4_, 2.7 mM KCl, 8.1 mM Na_2_HPO_4_, 10 mM EDTA) and filtered at 70 μm to yield individual cells. Two different comet methods were used to process the BAL cells/liver tissue and the lung tissue samples.

DNA SB in liver and BAL cells were analyzed using a high throughput protocol allowing 48 samples per GelBond^® ^film, developed at the Norwegian Institute of Public Health (Gunnar Brunborg and Kristine Bjerve Gutzkow) within the COMICS EU Project as previously described [35]. The BAL cells were quickly thawed in a 37°C waterbath before being mixed with agarose. The freezing and thawing of the cells was validated not to have any effect on experimental outcome in a cell line and in primary lymphocytes with correlation coefficients (r) of 0.96-0.99. In the present experiment, the cell/agarose suspension was applied onto a GelBond^(R) ^film (7 μL per sample) with a multichannel pipette. Eight films were processed per electrophoresis, in two parallel electrophoresis tanks. Due to preparation time, the lysing procedure varied between 1-2 hours for samples in the present study (up to 3.5 hours). The high volume protocol allowed processing of all related samples on one film and reduced the variation caused by increased processing time and different electrophoreses.

The slides were inspected using a Leica LB fluorescence microscope at 400 × magnification with a 450-490 nm emission filter and an LP515 excitation filter. DNA damage was measured as comet tail length (TL) using the Kinetics image analysing system (version 3.0). To eliminate day-to-day variation, the data were normalized to the level in A549 cells included on each electrophoresis gel. The mean TL ± SEM (μm) for the 0 and 30 μM H_2_O_2 _exposed A549 cells were 45.85 ± 4.49 and 73.27 ± 3.14 for analysis of the liver samples and 45.13 ± 2.59 and 71.92 ± 3.13 for the analysis of the BAL cells.

DNA SB in whole lung were measured as the formation of SB and FPG sensitive sites as previously described [72]. The level of FPG sensitive sites was calculated as the differences in DNA damage between slides that had been treated with the FPG enzyme and buffer. The FPG enzyme was a gift from Professor Andrew Collins (University of Oslo, Norway). Scoring was accomplished by visual inspection of 100 nuclei and categorized using a five class scoring system (ranging from 0-400). The five class scoring system shows excellent correlation with computerised image analysis of the DNA migration [73]. We used this calibration of the comet assay endpoint because this provides better inter-investigator accuracy [74,75] and reduces the inter-laboratory variation in reported values of DNA damage by the comet assay [76-78]. The data were transformed into lesions/10^6 ^bp using a previously established gamma irradiation calibration curve [76]. All samples were coded prior to scoring and organized to block the effects of day of experiments. We used human lymphocytes, treated with 1 μM Ro19-8022 photosensitizer and UV-light as control samples as described previously [79]. This treatment generates predominantly FPG sensitive sites as compared to SB. The Ro19-8022 photosensitizer was a gift from F. Hoffman, La Roche, Basel, Switzerland. The mean lesion per bp ± SEM were in these reference control samples were 0.068 ± 0.002 SB/10^6 ^bp and 2.045 ± 0.002 FPG sensitive site/10^6 ^bp.

### Saa3 mRNA expression

RNA was prepared using the NucleoSpin 96 RNA kit (Macherey-Nagel, Germany). RNA from the entire left lung of each mouse or a piece of liver tissue was prepared by lysing the tissue in 2 ml RLT buffer, while vigorously disrupting the sample with a Tissuelyser (Qiagen, Denmark) with a 5 mm stainless steel bead for 2 × 60 seconds and run through a QIAshredder (Qiagen, USA). The rest of the purification was performed as described by the manufacturer. cDNA was prepared using TaqMan reverse transcription reagents (Applied Biosystems, USA) as described by manufacturer.

*Saa3 *mRNA expression levels were determined as described previously [59].

### Statistics

The data on DNA damage, *Saa3 *mRNA expression and total number of cells in BAL fluid were analysed using a two-factor ANOVA analysis with the day of sacrifice and dose as categorical variables. We assessed differences in the BAL fluid cell composition using a one-factor ANOVA for each day of sacrifice, as well as by non-parametric Kruskal-Wallis or Mann-Whitney tests. The same significant effects were found in both parametric and non-parametric tests. The results on the number of cellular subsets of the BAL fluid were analyzed as log-transformed for macrophages and epithelial cells. Some samples of BAL fluid did not contain neutrophils, lymphocytes and eosinophils; we added the mean level of cells from the control group to all results to avoid problems with log-transformation of zero. Associations between the level of PMN and FPG sensitive sites in lung tissue were assessed by linear regression analysis with or without adjustment for effect of dose and time point. In all tests, the level of significance was 5%. The analyses were performed in Statistica version 5.5 for Windows (StatSoft Inc. (1997), USA).

## Competing interests

The authors declare that they have no competing interests.

## Authors' contributions

JAB, HW, UBV, CLY, NRJ, SL, and ATS contributed to the project idea and design. Exposures, BAL cell counts and BAL and liver comet assays were carried out by NRJ and ATS. JAB carried out comet assays and FPG modified comet assays on lung and prepared the manuscript. Particle characterization was done by KAJ. Endotoxin testing was done by AMM. JSL performed the *Saa3 *RT-PCR analyses. PM carried out statistical analyses of all data. All authors contributed to, examined and approved the final manuscript.
